# Individual differences in environmental wellbeing and pro-environmental behaviors explained by self-control

**DOI:** 10.3389/fpsyg.2023.1088682

**Published:** 2023-04-21

**Authors:** Camilla Strömbäck, Emma Lindkvist, Daniel Västfjäll

**Affiliations:** ^1^JEDI Lab, Division of Economics, Department of Management and Engineering, Linköping University, Linköping, Sweden; ^2^Division of Energy Systems, Department of Management and Engineering, Linköping University, Linköping, Sweden; ^3^JEDI Lab, Division of Psychology, Department of Behavioral Sciences and Learning, Linköping University, Linköping, Sweden; ^4^Decision Research, Eugene, OR, United States

**Keywords:** environmental wellbeing, pro-environmental behavior, self-control, political orientation, survey

## Abstract

Climate change is an increasing problem, with more extreme weather conditions and rising temperatures. To fulfill the temperature goals of the Paris agreement a societal change is needed, a change that requires a shift of lifestyle from all of us. If we want to change our behaviors to more sustainable ones, we need to sacrifice substantial things today to improve a future, which often seems distant and abstract. People with high level of self-control have been shown to have a better ability to visualize future events, which makes self-control an interesting trait to look at in relation to pro-environmental behavior. The aim of this study was to examine how self-control correlates with environmental well-being and environmental behavior. An internet-based survey was sent to a representative Swedish sample (*n* = 602). The respondents were asked to fill out a newly developed scale measuring their anxiety and security regarding environmental matters (environmental wellbeing), as well as indicate how often they engage in six different pro-environmental behaviors (e.g., turning lights off when leaving the room). Additionally, data on the respondents’ gender, age, political orientation, and self-control was collected. Our results suggest a positive correlation between self-control and environmental wellbeing and a weaker, but still positive, correlation between self-control and some pro-environmental behaviors. Additionally, respondents who identified themselves as politically left had lower environmental wellbeing, while men had higher environmental wellbeing, but behaved less environmentally friendly. Thus, our results suggest that political orientation was a better predictor of sound environmental behavior than subjective self-control was.

## Introduction

The emission of greenhouse gases to the atmosphere, caused by human activities, such as burning of fossil fuels and deforestation, is increasing. The increase of CO_2_, as well as other greenhouse gases such as CH_4_ and N_2_O, have led to an increase in the global surface temperature (IPCC, 2021). The last four decades have each been warmer than any decade before, since at least 1850. In 2015, 196 parties adopted the Paris Agreement, which is a legally binding agreement to limit global warming to well below 2 degrees Celsius above preindustrial levels, and preferably to only 1.5 degrees above (UNFCCC, 2015). To fulfill the goals of the Paris agreement a societal change is needed, a change that requires new ways of living. Many of us could, rather easily, make more environmentally friendly choices in our everyday life, and even though we are aware of this, and even though many of us want to change, we do not act accordingly. We overuse the resources of the planet, and thus accelerate the climate change.

Climate change does not only affect our physical living conditions but may also affect our mental health, both directly, e.g., when being victim to an extreme weather event, and indirectly, by creating distress and anxiety about the future (Fritze et al., 2008). Worry and anxiety caused by global warming, or other effects of climate change, is an increasing problem (Arcanjo, 2019) and the negative emotions rising from climate change do affect our general wellbeing (Stanley et al., 2021). However, the emotional response to climate change is a complex construct and may not only involve negative emotions. In certain contexts, climate change can be connected to empowerment rather than anxiety or worry (Verplanken et al., 2020). Understanding the determinants of people’s environmental wellbeing and pro-environmental behavior can help us design policies against climate change without adding unnecessary anxiety or distress. To minimize the negative emotions connected to climate change is not only important from a public health perspective, but also from a policy effectiveness perspective as eco-anxiety may lead to inaction and lower likelihood to join the cause (Verplanken et al., 2020; Stanley et al., 2021).

Previous research has found that political orientation and ideology correlate with environmental concern (Cruz, 2017) and that younger people show more cognitive involvement in environmental issues and have lower environmental well-being (Clayton and Karazsia, 2020). Motivation and political orientation have also been frequently used to explain who is acting in a pro-environmental way (Gromet et al., 2013; Wolsko et al., 2016; Panno et al., 2018). However, motivation and intentions, solely, are not enough to implement good environmental behavior. Many of the decisions we make that affect the environment, such as how to travel to work or what to eat for dinner, are made frequently and in a stable context, which means that they are strongly driven by habits (Klöckner, 2013). People with higher levels of self-control are better at implementing good habits to avoid situations in which they are tempted to act in contrary to their long-term goals (Carden and Wood, 2018). Additionally, it has been shown that people with higher levels of self-control have fewer psychological and emotional problems, including general anxiety (Tangney et al., 2004). In this study we want to investigate if the observed correlations between self-control and sound behaviors in other areas of life, as well as the positive correlation between self-control and higher wellbeing, also translate into the environmental domain. Thus, the aim of this study is to examine how self-control correlates with environmental wellbeing and environmental behavior, while controlling for political orientation, age, and gender.

Although the effect of self-control has been exhaustively researched within many areas, the correlation between environmental wellbeing and self-control has, to our knowledge, not been studied before. Regarding environmental behavior, previous research has found that people who failed to take the actions they thought to be reasonable and needed, to save the environment, were more likely to fail when trying to close the intention-behavior gap in other areas of life, such as the health domain (Redondo and Puelles, 2017). People high on trait self-control were also more likely to act in line with their environmental attitudes, which means that trait self-control was a good predictor of people reaching their long-term environmental goals (Wyss et al., 2022). Additionally, self-control has been shown to positively correlate with desirable behaviors in several other areas of life, including health (Moffitt et al., 2011), wealth (Strömbäck et al., 2017; Biljanovska and Palligkinis, 2018), and educational attainment (Mischel et al., 1989).

## Method

During spring 2021, an online survey^1^ was created and sent out to a representative sample of the Swedish adult population (aged 18–81). In total, 602 respondents (52% men, mean age 49 years, SD_age_ = 18.55) received a small monetary compensation for completing the survey, which was administrated by Origo Group. In the survey, information about the respondents’ self-assessed environmental behavior, environmental wellbeing, self-control, and political orientation was collected. On the question: “Do you identify yourself as politically left or politically right?,” 213 out of 602 respondent (35%) identified themselves as politically left and 268 respondents (45%) as politically right. In the regression models, political orientation was treated as a dichotomous variable taking either the value politically left or *not* politically left (including both the answers “politically right” and “I do not know”).

### Environmental wellbeing

The items used to measure environmental wellbeing were adapted from the Financial Anxiety Scale (Fünfgeld and Wang, 2009) and the Financial Security Scale (Strömbäck et al., 2017) to fit the environmental context. In the current context we operationalize environmental well-being broadly as a person’s feelings about their decisions and impact on the environment as well as a sense that the environment will be able to support the person’s current and future well-being. We acknowledge that environmental well-being theoretically is a much broader concept, including the impact of actions of other individuals, organizations and governments. The main reason that we opted for this narrower and preliminary definition of environmental well-being is twofold: (1) there is currently no consensus definition of the term environmental well-being, and (2) we wanted to keep the format and scope of our well-being measure similar to that used by us and others to examine financial well-being (Strömbäck et al., 2017, 2020). The participants were asked to indicate on a five-point Likert scale how well each statement corresponded to their own situation: five indicating that the participant agreed completely with the statement and one indicating that the participant did not agree at all with the statement. Table 1 shows all statements, their observed range, mean value and standard deviation. Items 1–4 were reversed before the mean value of the Environmental wellbeing scale was calculated. Hence, a higher score on the Environmental Wellbeing Scale indicates higher levels of environmental wellbeing. With a McDonald’s omega of 0.67, the scale shows reasonable high internal consistency.

**Table 1:** Summary statistics of the environmental wellbeing scale.

		Mean	SD	Observed range
**Environmental wellbeing scale**				
1	I get unsure by the lingo of climate and environmental experts	2.84	1.19	1–5
2	I am anxious about making decisions that could affect the environment	2.76	1.22	1–5
3	I tend to postpone decisions that could affect the environment as long as possible	2.64	1.26	1–5
4	After making a decision that could affect the environment, I am anxious whether I was right or wrong	2.28	1.14	1–5
5	When thinking about the environment, I feel secure in my current situation	3.27	1.08	1–5
6	When thinking about the environment, I feel confident about my future	2.49	1.15	1–5
7	I feel confident that the environment on Earth will be sufficiently good to support me, no matter how long I live	2.88	1.33	1–5
Environmental wellbeing, average		3.16	0.67	1.29–5

### Environmental behavior

The six pro-environmental behaviors measured in the survey were chosen from different subcomponents of the General Ecological Behavior Scale (Kaiser et al., 2003). Therefore, we will not treat them as a scale but analyze them separately. An advantage with looking at them separately is that we can distinguish if the respondents were more likely to act pro-environmentally in some areas of life rather than in others (Kaiser, 1998).

The respondents were asked to indicate on a 5-point Likert scale (1 = not at all, 5 = completely) to what extent each statement corresponded to their own behavior. As can be seen in Table 2, the respondents reported that they to a great extent engage in all six pro-environmental behaviors. Especially item 5 and 6, i.e., bringing empty bottles to the recycle bin and leaving a picnic site as clean as it was upon arrival, had extremely skewed distributions.

**Table 2:** Summary statistics of the environmental behaviors.

		Mean	SD	Observed range
**Environmental behaviors**				
1	When being the last person leaving a room, I turn the light off	4.17	0.87	1–5
2	I ride a bicycle or take public transportation to work or school	3.21	1.56	1–5
3	When possible, I buy products in refillable packages (e.g., spices and soap)	3.72	0.94	1–5
4	I buy products with eco-labels	3.19	0.91	1–5
5	I bring empty bottles to a recycling bin	4.71	0.72	1–5
6	After a picnic, I leave the place as clean as it was originally	4.85	0.53	1–5

Table 3 shows the correlations between the different pro-environmental behaviors and environmental wellbeing. All pro-environmental behaviors were positively, but not strongly, correlated with each other, which indicate that some respondents act generally more pro-environmental than others. We also find small, negative correlations between all pro-environmental behaviors and environmental wellbeing.

**Table 3:** Correlations between the dependent variables and self-control.

	Turn light off	Good transportation to work	Refill packages	Organic products	Recycle cans	Clean after picnic	Environ-mental well-being	Self-control
Turn light off	1.00							
Good transportation to work	0.10	1.00						
Refill packages	0.21	0.11	1.00					
Organic products	0.11	0.10	0.21	1.00				
Recycle cans	0.17	0.03	0.23	0.09	1.00			
Clean after picnic	0.27	0.04	0.15	0.09	0.36	1.00		
Environmental well-being	0.00	−0.12	−0.12	−0.21	−0.07	−0.04	1.00	
Self-control	0.08	0.00	0.11	0.05	0.12	0.09	0.20	1.00
Observations	602							

### Self-control

To assess the respondents’ self-control, we used the same scale as Strömbäck et al. (2017), which is a combination of the Brief Self-Control Scale (Tangney et al., 2004) and the Short Term Orientation Scale (Antonides et al., 2011). Once again, the respondents were asked to indicate on a 5-point Likert’s scale how well each statement corresponded to themselves (1 = not at all, 5 = totally agree). Table 4 shows the nine items, their observed range, mean value, and standard deviation. The internal consistency of the self-control scale was good (McDonald’s *ω* = 0.78).

**Table 4:** Summary statistics of the self-control scale.

Self-control scale		Mean	SD	Observed range
**Brief self-control scale**				
1	I have a hard time breaking bad habits	3.03	1.16	1–5
2	I get distracted easily	2.84	1.22	1–5
3	I’m good at resisting temptation	3.31	1.08	1–5
4	I do things that feel good in the moment but regret later on	2.37	1.14	1–5
5	I often act without thinking through all the alternatives	2.19	1.13	1–5
**Short term orientation scale**				
6	I only focus on the short term	2.04	1.07	1–5
7	The future will take care of itself	2.94	1.17	1–5
8	I live more for the day of today than for the day of tomorrow	2.22	1.14	1–5
9	My convenience plays an important role in the decisions I make	3.42	1.06	1–5
Self-control, average		3.36	0.67	1.44–5

Item 1, 2, 4, 5, 6, 7, 8, and 9 were reversed before calculating the aggregated mean value of self-control.

## Results

### Self-control and environmental wellbeing

**Figure 1:**
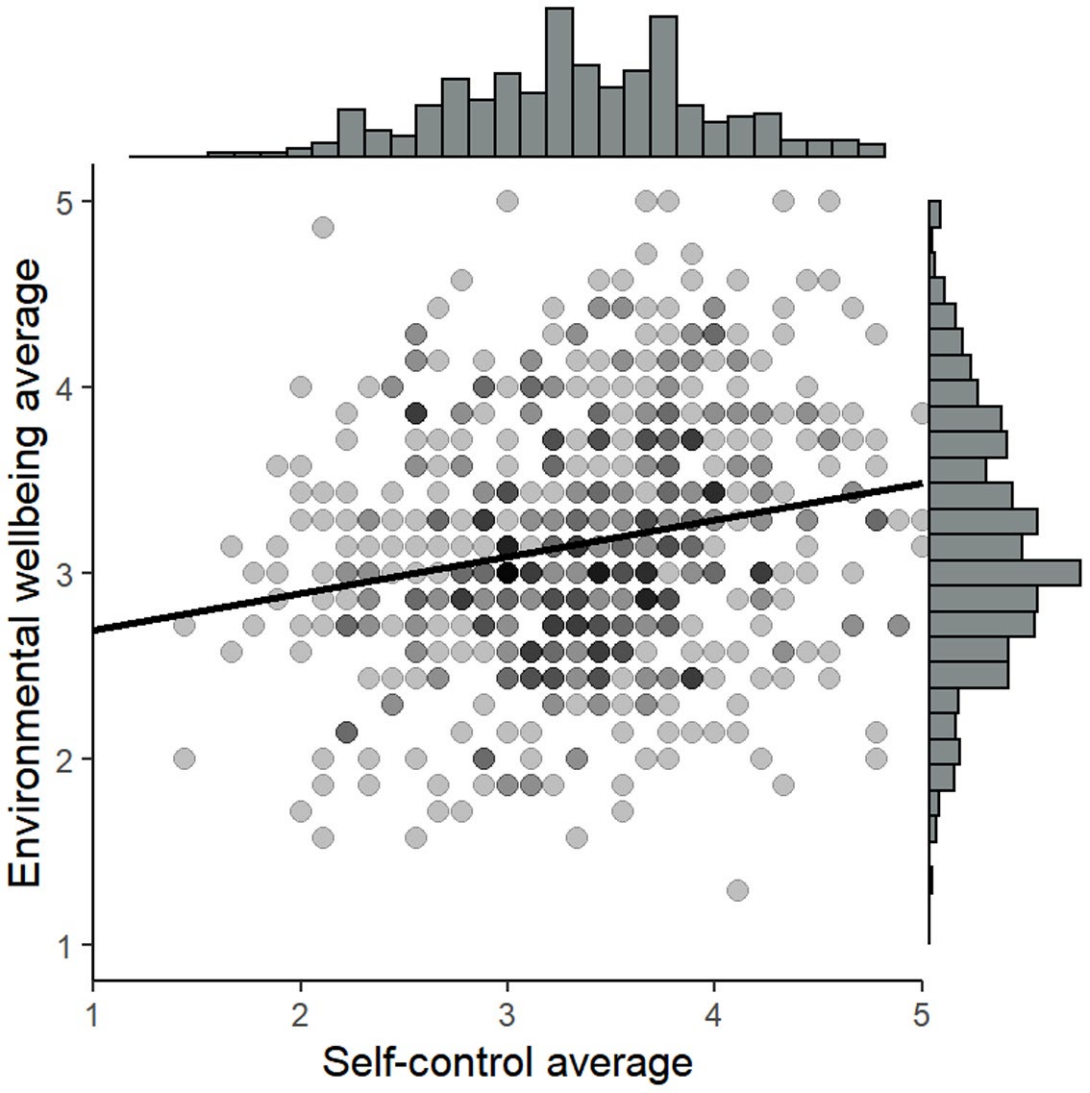
Scatterplot of the correlation between environmental wellbeing and self-control. The *y*-axis shows the average of the environmental wellbeing scale and the *x*-axis shows the respondents’ self-control.

Figure 1 is a scatterplot showing the correlation between self-control and environmental wellbeing. The *y*-axis represents each respondent’s average on the environmental wellbeing scale, while the *x*-axis shows their average on the self-control scale. The colors of the dots correspond to the numbers of respondents it represents, with darker dots representing more individuals. The slope of the trend line suggests a positive correlation between self-control and environmental wellbeing. The Pearson correlation between environmental wellbeing and self-control is *r*(600) = 0.20, *p* < 0.001, which according to Gignac and Szodorai (2016) corresponds to a medium effect size.

The next step is to analyze if the observed positive correlation between self-control and environmental wellbeing persists when other variables are included in the analysis. Table 5 shows the results of an OLS-regression, with environmental wellbeing as the dependent variable and self-control, political orientation, age, and gender as independent variables. Self-control was positively, and statistically significantly correlated with environmental wellbeing, which means that the respondents who reported higher levels of self-control on average also reported higher environmental wellbeing. Apart from that, we can observe that men had higher environmental wellbeing than women and that respondents who reported their political orientation to be to the left had lower environmental wellbeing.

**Table 5:** Environmental wellbeing as a function of self-control, political orientation, and control variables.

(1) Environmental wellbeing	
Self-control	0.217***
	(0.136–0.298)
Political left	−0.204***
	(−0.310 – −0.098)
Age	0.002
	(−0.001–0.004)
Men	0.322***
	(0.221–0.424)
Observations	598
R-squared	0.126

The regression is an OLS-model. Robust confidence intervals in parentheses. ***p < 0.01, **p < 0.05, *p < 0.1.

### Self-control and pro-environmental behavior

**Figure 2:**
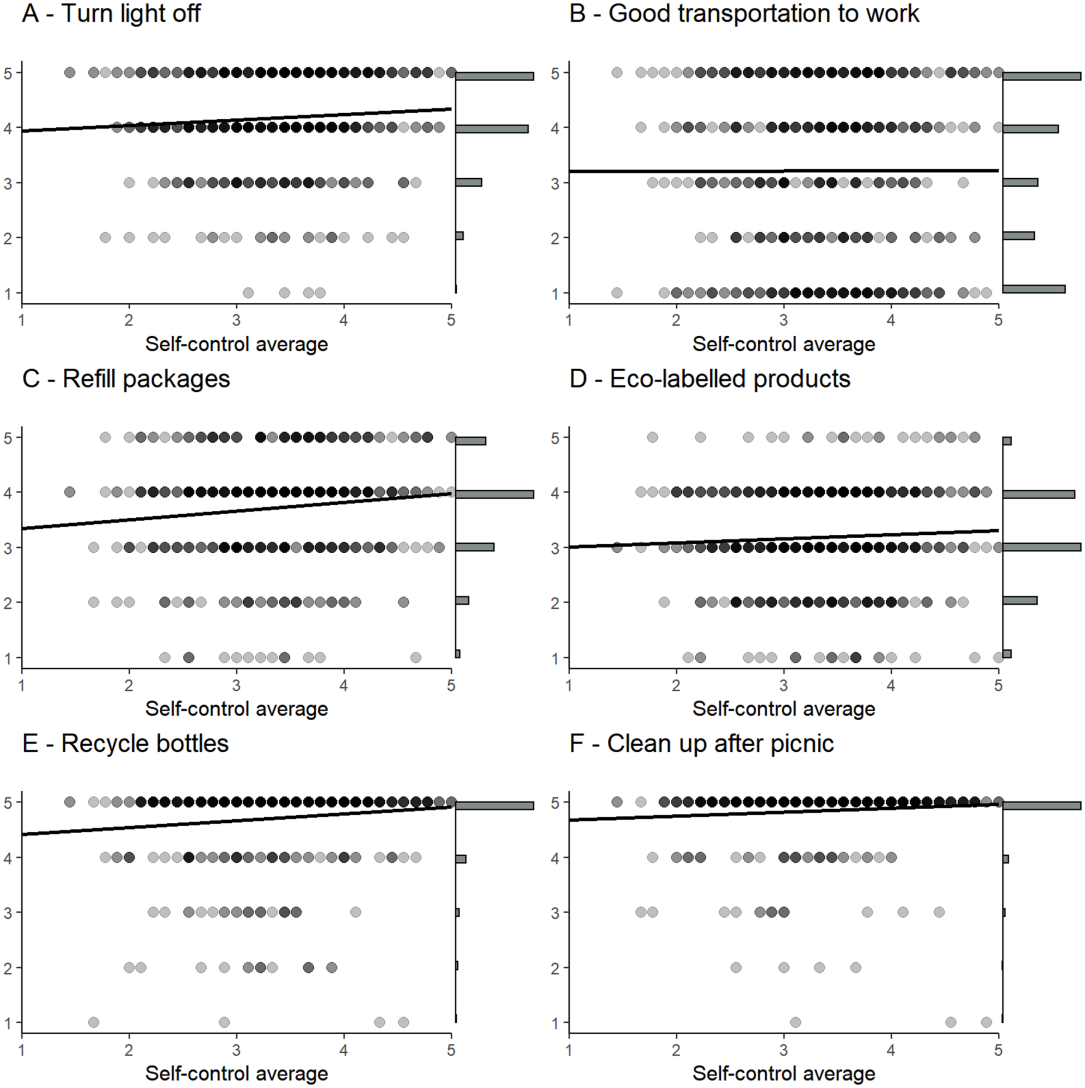
Scatterplots of the pro-environmental behaviors and self-control. The *y*-axes show how often the respondent engage in the different behaviors, while the *x*-axes show the respondents’ self-control. Scatterplott **(A-F)** correspond to the six differnt pro-environmental behaviors.

As the pro-environmental behaviors will be investigated separately, Figure 2 consists of six different scatterplots, each representing one pro-environmental behavior. The *y*-axis in each graph shows how often the respondent engage in each behavior (on a scale from 1 = never to 5 = always), and the *x*-axis represents the results on the self-control scale. Once again, the colors of the dots indicate how many respondents it represents, with darker colors representing a higher number. On the right side of each scatterplot is a histogram showing the distribution of the pro-environmental behavior in question.

Noticeable is that the pro-environmental behaviors are not normally distributed, but in most cases skewed toward higher numbers. The bottom two behaviors are the most problematic ones; 82 percent of the respondents reported that they always bring empty bottles to the recycle bins and 90 percent that they always leave a picnic site as clean as it was upon arrival. Taking all six scatterplots into consideration, we can observe a small positive correlation between self-control and pro-environmental behaviors. When including the results from Table 3, we can conclude that the positive correlation between self-control and the pro-environmental behaviors ranges from 0.001 to 0.12, which according to Gignac and Szodorai (2016) would be categorized as non-existing to small effect sizes.

As a next step we ran OLS-regressions with the six pro-environmental behaviors as dependent variables, and included self-control, political orientation, age, and gender as independent variables. Table 6 shows the results of the OLS-regressions. We found a positive correlation between self-control and the likelihood to turn the lights off when leaving a room. However, self-control did not statistically significantly correlate with any of the other pro-environmental behaviors. Respondents who identified themselves as politically left were less likely to turn the lights off when leaving the room, but more likely to engage in several other pro-environmental behaviors. In the cases where we observe a gender effect, men report worse pro-environmental behaviors than women. So, although we do not observe any strong effects of self-control, we have power enough to detect existing effects, which indicate that self-control may not be the most important determinant when it comes to predicting these pro-environmental behaviors.

**Table 6:** Pro-environmental behaviors as functions of self-control, political orientation, and control variables.

	(1)	(2)	(3)	(4)	(5)	(6)
	Turn light off	Good transportation to work	Refill packages	Eco-labeled products	Recycle bottles	Clean up after picnic
Self-control	0.119**	0.093	0.116*	0.041	0.021	0.017	(0.012–0.226)	(−0.095–0.280)	(−0.004–0.235)	(−0.072–0.155)	(−0.062–0.104)	(−0.058–0.093)
	Political left	−0.194**	0.326***	0.113	0.218***	0.070	0.123***
(−0.342 – −0.046)		(0.082–0.571)	(−0.037–0.263)	(0.072–0.364)	(−0.040–0.179)	(0.048–0.198)
Age	−0.002	−0.018***	0.002	0.002	0.009***	0.004***
	(−0.006–0.002)	(−0.024 – −0.012)	(−0.002–0.007)	(−0.002–0.005)	(0.0061–0.013)	(0.002–0.007)
Men	−0.099	−0.453***	−0.295***	−0.257***	−0.104*	−0.056
	(−0.239–0.042)	(−0.698 – −0.209)	(−0.444 – −0.146)	(−0.401 – −0.112)	(−0.209–0.001)	(−0.135–0.022)
Observations	598	598	598	598	598	598
R-squared	0.022	0.082	0.042	0.038	0.074	0.043

All regressions are OLS models. Robust confidence intervals in parentheses. ***p < 0.01, **p < 0.05, *p < 0.1.

As we observe clear ceiling effects for several of the pro-environmental behaviors, the results of the OLS-regressions were controlled with tobit regressions^2^. When looking at the first four behaviors (model 1–4), the results from OLS and tobit regressions are similar. The only noticeable difference is that we observed a statistically significant (positive) effect of self-control in model 3 when using tobit. The results of model 5 and 6 differ more between the OLS or tobit regressions. However, these variables suffer from such strong ceiling effects, that neither of the results are strong evidence for anything.

## Discussion

This paper set out to examine how self-control correlates with environmental wellbeing and a set of pro-environmental behaviors. This was done by the distribution of a survey to a fairly representative sample of the Swedish population. The results indicate that respondents who reported higher levels of self-control had higher environmental wellbeing and, to some extent, were more likely to engage in pro-environmental behaviors. Thus, our findings are consistent with existing literature on self-control which have found that higher self-control is positively correlated with financial wellbeing (Strömbäck et al., 2017), general wellbeing (Tangney et al., 2004; Hofmann et al., 2014) and several desirable behaviors in other areas of life (De Ridder et al., 2012).

An important note is that the results regarding pro-environmental behavior might be affected by the observed ceiling effects in these variables. There are especially two of the six measured behaviors (returning bottles and cleaning up after picnic), where we observed strong ceiling effects. Given that 86 percent of all plastic bottles in Sweden were returned in year 2020 (SCB, 2021), this is not a surprising finding. However, in a recent study by Panwanitdumrong and Chen (2021) using the same item regarding cleaning up after a picnic, the variation was much larger, with a mean of 2.97 on a 5-point Likert scale (SD = 1.03). If these observed differences are due to cultural differences or to social desirability bias among our respondents is hard to know, but the results for this item may, in our sample, be inflated.

Apart from the effects of self-control on environmental wellbeing and pro-environmental behaviors, we observe that men had higher environmental wellbeing and were less likely to behave pro-environmentally. Earlier studies have shown similar results, in terms of women being more likely to act pro-environmentally, to have more climate anxiety (Clayton and Karazsia, 2020), and suffer higher risk of general anxiety (McLean and Anderson, 2009). We can also observe that respondents considering themselves as politically left had lower environmental wellbeing and were more likely to act pro-environmentally. As the effect of political ideology may be country and context specific (Flores et al., 2022; Van Bavel et al., 2022), more research about the impact of political orientation is needed. Further studies should also consider assessing political orientation as a continuous, rather than binary, variable. Still, our results are in line with those of a meta-analysis performed by Cruz (2017), which showed that people identifying themselves as political left were more likely to have more environmental concerns.

The environmental wellbeing scale was adapted from the Financial Security scale (Strömbäck et al., 2017) and the Financial Anxiety scale (Fünfgeld and Wang, 2009). Like for financial wellbeing, people felt relatively secure in their current environmental situation. However, an important difference between financial wellbeing and environmental wellbeing was the respondents’ view of their long-run security. The respondents’ of the financial study stated that they felt more confident about their financial future than about being able to support themselves financially throughout their life (Strömbäck et al., 2017), while the respondents’ of this study felt less secure about the future when considering the environment than about whether the Earth will be able to support them during their lifetime. This could possibly reflect that environmental wellbeing is affected by concerns about the environment for future generations and the actions of other people and organizations, while financial wellbeing is more affected by concerns related to our own lifetime and more under our own control. The intergenerational nature of the climate crisis is often viewed as a barrier to change, but it has also been put forward as a possible solution. Zaval et al. (2015) showed in experiments that making the participants’ legacy more saliant increased their pro-environmental behaviors and intentions. Moreover, while the goal for financial well-being is to reduce financial anxiety and increase financial security, it is likely that that pro-environmental behaviors are both motivated and demotivated by environmental anxiety (“my decisions do/do not matter”) and security (“the future environment is not going to be secure”). Our preliminary definition of environmental well-being was adopted to make comparisons with financial well-being simpler but comes at the cost of a narrow operationalization of the construct. Future research should expand the measurement of environmental well-being to include feelings about your own actions, other’s actions and their combination (collective action). A more exhaustive measure of environmental well-being may also correlate differently with behavioral intentions, self-reported pro-environmental behaviors and self-control.

To adopt to a more sustainable lifestyle, we need to transitionally substitute old environmentally harmful behaviors with more environmentally friendly habits. This might sound fairly easy, but changing one’s habits are generally difficult (Carden and Wood, 2018). And although people with higher level of self-reported self-control are better at implementing habits to avoid situations where they need to inhibit impulses that go against their long-term goals (De Ridder et al., 2012; Galla and Duckworth, 2015), we only observe a small positive effect of self-control on pro-environmental behavior. At this stage, we can only hypothesize about the underlying reasons for this. If the motivation to live more environmentally friendly is not strong enough, the increased ability among people with higher levels of self-control to act in accordance with their long-term goals (Mischel et al., 1989; Pyone and Isen, 2011) may not increase pro-environmental behaviors to a great extent. Another possible explanation is that the average person lacks the general knowledge of how we most effectively switch to more environmentally friendly lives. And although information alone is not enough to change habits (Casagrande et al., 2007), knowledge is still needed to implement the right changes. As we need to change our habits to be able to fulfill the Paris Agreement, future studies should look closer into the underlying mechanisms for habitual change that could lead to a more sustainable lifestyle. As some of the crucial changes we need to implement will include making personal sacrifices for a greater good, the possible correlation between prosocial personality traits, pro-environmental behaviors, and environmental wellbeing might also be of interest.

## Data availability statement

The datasets presented in this study can be found in online repositories. The names of the repository/repositories and accession number(s) can be found below: https://osf.io/dgxfy/?view_only=b3bf403cf67d42579748bc83072cdaac.

## Ethics statement

Ethical review and approval was not required for the study on human participants in accordance with the local legislation and institutional requirements. The patients/participants provided their written informed consent to participate in this study.

## Author contributions

DV contributed to conception and design of the study. DV organized the data collection. CS performed the statistical analysis. CS and EL were responsible for writing the manuscript. DV came with comments on the manuscript. All authors contributed to manuscript revision, read, and approved the submitted version.

## Funding

This research was funded by the Formas – The Swedish Research Council for Environment, Agricultural Sciences and Spatial Planning. The funders had no role in study design, data collection, and analysis, decision to publish, or preparation of the manuscript.

## Conflict of interest

The authors declare that the research was conducted in the absence of any commercial or financial relationships that could be construed as a potential conflict of interest.

## Publisher’s note

All claims expressed in this article are solely those of the authors and do not necessarily represent those of their affiliated organizations, or those of the publisher, the editors and the reviewers. Any product that may be evaluated in this article, or claim that may be made by its manufacturer, is not guaranteed or endorsed by the publisher.

## Footnotes

^1^The survey and data collection were part of a master thesis (Lilliesköld, 2021). Supervised by the last author.

^2^The results from the tobit regressions are found in the Supplementary Appendix.
