# Supplementary material for: Individual differences in environmental wellbeing and pro-environmental behaviors explained by self-control
Source: Front Psychol. 2023 Apr 21;14:1088682. doi: 10.3389/fpsyg.2023.1088682 (PMC10160623; doi:10.3389/fpsyg.2023.1088682)
Supplement: Supplementary file 1 [file Table_1.DOCX]

Table A1. Tobit regression on pro-environmental behavior.

|  | b1 | | b2 | | b3 | | b4 | | b5 | | b6 | |
| --- | --- | --- | --- | --- | --- | --- | --- | --- | --- | --- | --- | --- |
|  | Coeff (SE) | Marginal  Effects | Coeff (SE) | Marginal  Effects | Coeff (SE) | Marginal  Effects | Coeff (SE) | Marginal  Effects | Coeff (SE) | Marginal  Effects | Coeff (SE) | Marginal  Effects |
| sc_avg | 0.23^**^  (0.09) | 0,14 | 0.21  (0.20) | 0,11 | 0.16^**^  (0.07) | 0,13 | 0.05  (0.07) | 0,05 | 0.27^***^  (0.07) | 0,04 | 0.52^***^  (0.07) | 0,04 |
| male | -0.2*  (0.12) | -0,12 | -0.89^***^  (0.26) | -0,45 | -0.37^***^  (0.10) | -0,31 | -0.27^***^  (0.10) | -0,26 | -0.66^***^  (0.10) | -0,1 | -0.71^***^  (0.10) | -0,06 |
| age | -0.004  (0.003) | -0,002 | -0.04^***^  (0.01) | -0,02 | 0.002  (0.003) | 0,002 | 0.002  (0.003) | 0,002 | 0.05^***^  (0.003) | 0,01 | 0.04^***^  (0.003) | 0,003 |
| pol_left | -0.35^***^  (0.13) | -0,21 | 0.70^***^  (0.27) | 0,35 | 0.12  (0.10) | 0,1 | 0.24^**^  (0.10) | 0,23 | 0.35^***^  (0.10) | 0,05 | 1.20^***^  (0.10) | 0,1 |
| logSigma | 0.31^***^  (0.04) |  | 1.07^***^  (0.05) |  | 0.12^***^  (0.03) |  | -0.04  (0.03) |  | 0.88^***^  (0.03) |  | 1.05^***^  (0.03) |  |
| Constant | 4.21^***^  (0.33) |  | 4.81^***^  (0.72) |  | 3.34^***^  (0.26) |  | 2.99^***^  (0.26) |  | 4.17^***^  (0.26) |  | 5.34^***^  (0.26) |  |
| Observations | 598 | 598 | 598 | 598 | 598 | 598 | 598 | 598 | 598 | 598 | 598 | 598 |
| Log Likelihood | -799,93 |  | -982,25 |  | -868,93 |  | -828,24 |  | -396,47 |  | -252,92 |  |
| *Note:* Tobit regressions on pro-environmental behaviors. ^*^p<0.1; ^**^p<0.05; ^***^p<0.01 | | | | | | | | | | | | |
